# LevioSAM: fast lift-over of variant-aware reference alignments

**DOI:** 10.1093/bioinformatics/btab396

**Published:** 2021-05-25

**Authors:** Taher Mun, Nae-Chyun Chen, Ben Langmead

**Affiliations:** Department of Computer Science, Johns Hopkins University, Baltimore, MD 21218, USA; Department of Computer Science, Johns Hopkins University, Baltimore, MD 21218, USA; Department of Computer Science, Johns Hopkins University, Baltimore, MD 21218, USA

## Abstract

**Motivation:**

As more population genetics datasets and population-specific references become available, the task of translating (‘lifting’) read alignments from one reference coordinate system to another is becoming more common. Existing tools generally require a chain file, whereas VCF files are the more common way to represent variation. Existing tools also do not make effective use of threads, creating a post-alignment bottleneck.

**Results:**

LevioSAM is a tool for lifting SAM/BAM alignments from one reference to another using a VCF file containing population variants. LevioSAM uses succinct data structures and scales efficiently to many threads. When run downstream of a read aligner, levioSAM is more than 7 times faster than an aligner when both are run with 16 threads.

**Availability and implementation:**

Software Package: https://github.com/alshai/levioSAM, Experiments: https://github.com/langmead-lab/levioSAM-experiments

**Supplementary information:**

Supplementary data are available at *Bioinformatics* online.

## 1 Introduction

Most analyses of sequencing datasets start by aligning reads to a linear reference genome. However, the use of a single linear reference leads to a phenomenon called reference bias—the tendency to produce incorrect alignments or entirely miss them for reads containing non-reference alleles. Some methods for reducing bias by substitute alternate alleles into one or more linear references in order to create *variant-aware* references.

One such approach is to use the major allele reference (Dewey *et al.*, 2011), which substitutes common alternate alleles for rarer reference alleles. A second one is to specialize the reference using alleles specific to an individual (Rozowsky *et al.*, 2011). A third approach is to use multiple population-specific references, which improves accuracy and reduces reference bias (Chen *et al.*, 2021). In these cases, variant-aware references use the original reference as a backbone; i.e. all references can be constructed from a linear reference together with a Variant Call Format (VCF) file describing differences from that reference. While it is also useful to consider alternate *assemblies*, where the assemblies may lack a common coordinate system, we do not consider that scenario here.

Variant-aware alignment methods require a step that translates, or ‘lifts over’ the alignments from the variant-aware references back to the standard reference coordinate system. This problem has been addressed to some degree in prior tools like UCSC liftOver (Fujita *et al.*, 2011) and CrossMap (Zhao et al., 2014). For the variant-aware alignment scenarios described above, an ideal lift-over tool will: (i) translate both the alignment coordinates and the auxiliary information in a SAM/BAM record (Li *et al.*, 2009) such as the CIGAR string, MD tag and NM tag, (ii) retrieve coordinate conversion information from VCF files Li *et al.*, (2009), since this is the typical format used in large-scale population genetics studies (1000 Genomes Project Consortium *et al.*, 2015; Danecek *et al.*, 2011; Kaminow *et al.*, 2020), (iii) support simultaneous use of many threads, so that lift-over can complete quickly, preferably in only a fraction of the time required for the read alignment step.

While CrossMap partially supports lifting alignments in SAM/BAM format, it does not handle all relevant SAM fields and requires a ‘chain file’ to describe the coordinate transformation, which is more difficult to work with compared to a VCF. CrossMap also does not support multi-threading, making it a likely bottleneck in real analysis workflows.

We describe levioSAM, an efficient and scalable tool for lifting alignments from variant-aware reference to a target reference genome. LevioSAM supports lift-over from a variant-aware reference described by a VCF file and receives/delivers a SAM/BAM as input/output. In addition, levioSAM supports multi-threading, allowing it to scale to millions of reads. When compared to CrossMap, levioSAM produces identical alignment offsets, while also supporting CIGAR strings and SAM tags.

## 2 Approach

LevioSAM uses succinct data structures from the SDSL library (Gog *et al.*, 2014) to represent indel differences between the source and target references (Supplementary Note S1, Supplementary Fig. S1). As of now, levioSAM does not support forms of variation more complex than the SNVs and indels that can be readily represented in VCF files. Larger scale variants like re-arrangements and inversions are not supported. The succinct structures generated by levioSAM can be serialized on disk so they can be reused without recalculating differences. LevioSAM supports BAM/SAM input files and handles lift-over of all mandatory fields of a SAM record, including the alignment offset, CIGAR string, MAPQ and paired-read information. LevioSAM also supports the N CIGAR operation, allowing it to lift spliced alignments. Optionally, levioSAM will also lift the MD and NM SAM tags (Supplementary Note S2). Multithreading and piped input/output are supported as well, allowing levioSAM to run directly in tandem with aligners that write to standard output.

## 3 Results

A common variant-aware alignment scenario is to align first to a major-allele reference, then translate the alignments back to the standard reference. We evaluated the computational efficiency of levioSAM when lifting human alignments from a major-allele version of GRCh38 to the standard GRCh38 reference. We built the major-allele reference based on the 1000 Genomes Project call set (Lowy-Gallego *et al.*, 2019), producing variant-aware reference with 1 746 180 single-nucleotide variants and 252 781 indels with respect to GRCh38. We randomly sampled 10 million 150-bp Illumina reads from ERR3239334, a whole-genome sequencing dataset derived from the NA12878 individual. We used Bowtie 2 (Langmead and Salzberg, 2012) with default parameters to align sequencing reads to the major-allele GRCh38 reference.

**Fig. 1.**
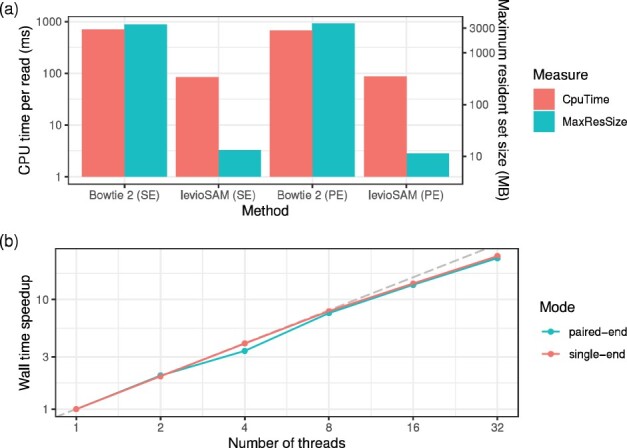
(**a**) Runtime of alignment and levioSAM using single-end (SE) and paired-end (PE) modes. Ten million 150-bp reads are processed using 16 threads. (**b**) Wall time speedup when using multiple threads. With one thread (1×), levioSAM uses 74.3 ms for a single-end read and 79.0 ms for a paired-end read segment. The gray dashed line represents the perfect scenario

LevioSAM used only a fraction of the computational resources used by Bowtie 2 during alignment (Fig. 1a) when both were run on the read set using 16 threads. When comparing the CPU time we found that levioSAM runs more than 8 times faster than Bowtie 2 for single-end reads (83.6 ms versus 699.3 ms per read), and more than 7 times faster for paired-end reads (87.0 versus 673.8 ms per read). LevioSAM used less than 13 MB memory in both cases, a fraction of Bowtie 2’s 3 GB footprint.

LevioSAM scaled efficiently with added threads. When run with 32 threads, it completed 25 times faster than when using 1 thread and when lifting unpaired alignments, and 23.9 times faster for paired-end alignments (Fig. 1b). LevioSAM’s peak memory footprint was 54% higher for 32 threads compared to a single thread, but peak memory never exceeded 16 MB.

We then compared the run-time and memory of levioSAM to that of CrossMap using the same set of reads. We did not compare to the common UCSC liftOver tool as it does not support SAM/BAM input files. We converted the major-allele VCF to a chain file for use with CrossMap. LevioSAM and CrossMap produced identical lift-over coordinates and mapping qualities for each alignment. LevioSAM accurately lifted all the CIGAR strings, but CrossMap does not support CIGAR string conversion, resulting in 1.09% of its CIGAR strings to be invalid after lift-over. CrossMap is faster than levioSAM when running on a single thread, but has a higher memory footprint. However, levioSAM, when run with 16 threads, was more than 8× faster than Bowtie 2, whereas CrossMap, which lacks multithreading, was 1.19× slower than Bowtie 2 (Supplementary Note S3). This highlights the importance of multi-threading in such tools.

We observed that lift-over of variant-aware alignments can improve read-alignment accuracy for workflows involving both Bowtie 2 and BWA-MEM (Li, 2013) (Supplementary Note S4).

Overall, levioSAM’s memory efficiency, speed and thread scaling allow it to fit in a typical read alignment workflow without creating a new bottleneck downstream of read alignment. These experiments were performed on a computer with a 2.2 Ghz Intel Xeon CPU (E5-2650 v4) and 515 GB memory.

## 4 Discussion

LevioSAM is an alignment lift-over tool for working with variant-aware references (references generated by substituting known variants into a reference genome). It supports SAM/BAM and VCF files and makes it easier to implement efficient workflows for read alignment to one or more customized reference genomes. LevioSAM is based on succinct data structures and scales well with multiple threads, making it computationally efficient to be integrated in alignment pipelines. LevioSAM has been applied to the problem of alignment to multiple population-specific genomes in the past (Chen *et al.*, 2021), and we provide a tutorial on how to integrate it with other common workflows that use variant-aware references (Supplementary Note S4). While levioSAM currently assumes that differences between the source and target references consist only of mismatches and indels, it will be important to support larger differences such as inversions or rearrangements in the future.

## Supplementary Material

btab396_Supplementary_Data
