# Supplementary material for: LevioSAM: fast lift-over of variant-aware reference alignments
Source: Bioinformatics. 2021 May 25;37(22):4243–5. doi: 10.1093/bioinformatics/btab396 (PMC9502237; doi:10.1093/bioinformatics/btab396)
Supplement: btab396_Supplementary_Data [file btab396_supplementary_data.pdf]

Supplementary Information for LevioSAM: Fast lift-over of variant-aware reference alignments

May 18, 2021

S1 Supplementary Notes

S1 LevioSAM implementation

LevioSAM is a C++ program that utilizes succinct data structures provided by the `sdsl-lite` 2.0 library [5] in conjunction with the SAM and VCF parsing tools provided by the `htslib` library [2]. Supplementary Figure S1 shows a visual representation of how levioSAM lifts over positions from a variant-aware reference to the original reference.

Briefly, the pairwise sequence alignment between the two references is represented as two bit-vectors, where a `1` in the first bit-vector represents an insertion with respect to the reference in the in alignment, and a `1` in the second bit-vector represents a deletion. To lift-over a position from the variant-aware reference, a `select0` query is performed on the second bit-vector to get the position in the over-all pairwise alignment. Using this value, a `rank0` query is performed on the first bit-vector to obtain the position within the reference genome. A lift-over from the reference to the variant-aware sequence can similarly be done by performing a select operation on the top sequence and then a rank operation on the bottom sequence.

S2 Lifting NM and MD tags

LevioSAM optionally supports updating commonly used SAM tags during lift-over. By enabling the `-m` option, levioSAM updates the `NM:i` (edit distance to the reference) and `MD:z` (string encoding mismatches and deleted reference bases) tags. LevioSAM applies functions from samtools v1.11 [11, 2] to perform the update, and thus `leviosam lift -m` and `samtools calmd` share the same logic.

Re-calculating the `NM:i` and `MD:z` tags require information from the reference, and thus we suggest users pre-sort the input SAM/BAM file when using `-m` to avoid huge computational overhead due to cache misses. Without sorting, lift-over the 10M 150-bp single-end datasets (as Sections S4 and S3) using levioSAM took 746,637 CPU seconds (46,698 wall clock seconds, or near 13 hours) to complete with 16 threads. When the dataset was pre-sorted prior to lift-over, levioSAM used 1,082 CPU seconds (94 wall clock seconds) to complete full lift-over with 16 threads.

S3 Comparison of levioSAM and CrossMap

LevioSAM and CrossMap [14] are the only two tools supporting liftover of aligned reads (in SAM/BAM format) to the best of our knowledge [8]. While UCSC liftOver [4] is commonly used for liftover purposes, it only supports the BED format and doesn't apply to our target applications. Both levioSAM and CrossMap accurately converts the mapping positions (POS field in the SAM format), but only levioSAM performs correct CIGAR string conversion. When using the optional `-m` command, levioSAM can additionally lift the `NM:i` and `MD:z` tags. CrossMap sorts the alignments as a by-product, whereas levioSAM defers that to downstream steps (such as `samtools sort`).

We used 10M 150-bp single-end reads, aligned using Bowtie 2, to assess the computational performance of levioSAM and CrossMap. We used default parameters for both tools unless additionally specified. When operating in single thread, CrossMap was 1.7x faster than levioSAM (527 vs. 819 seconds), but used 93x more memory (951 MB vs. 10 MB). When using 16 threads, thanks to the multithreading support of levioSAM, it is 8.6x as fast as CrossMap in wall clock time (61 vs. 529 seconds) and uses less memory (13 MB vs. 951 MB). We also observed that CrossMap was slower than Bowtie 2 in the 16-thread experiment (529 vs. 442 seconds). This likely makes CrossMap a bottleneck in variant-aware reference alignment pipelines, such as those using major-allele reference or personalized references (Section S4).

We would additionally like to point out that currently is it not straightforward to convert a VCF file to a chain file. In our experiment, we used `bcfttols consensus -c` to generate a chain file, but the provided source and target were opposite to what was defined by CrossMap. Thus, we used an `awk` script for conversion. See the commands below, or `https://github.com/alshai/levioSAM/blob/master/scripts/vcf2chain.sh`.

S4 Using levioSAM in an variant-aware reference genome alignment pipeline

Alignment using variant-aware reference genomes has been applied to various genomics applications to reduce reference bias, with different types of sequencing data. Chen et al. [3] and Pritt et al. [12] showed that using major-allele reference improved alignment accuracy using DNA sequences. Kaminow et al. [7] applied major-allele reference to improve RNA-Seq analysis. Yuan and Qin [13] showed that using diploid personalized reference genome reduced reference bias for detecting allele-specific expression using RNA-Seq data.

LevioSAM is designed to be integrated with applications based on major-allele reference and personalized reference. In the Results Section we showed that levioSAM is fast and memory-efficient compared to the computational cost of alignment. In this section, further we assessed the alignment accuracy when integrating variant-aware reference genomes with levioSAM.

We started the experiment by simulating 10M 150-bp paired-end reads using the mason simulator [6] from the chr21 of individual NA12878. We used genotypes from the 1000 Genomes Project [1] to generate the genome of NA12878. Both SNVs and indels were used. For simplicity, we only used the first haplotype. We then aligned the reads to the chr21 of GRCh38, major-allele reference and the NA12878 genome using Bowtie2 [9] and bwa-mem [10] (Tables S1 and S2). We showed that when switching using the major-allele reference improved alignment results in all tested measures (number of unaligned reads, fraction of correct alignments and fraction of correct reads). Alignment accuracy was further improved when using the personalized reference genome.

We provide a detailed tutorial to perform alignment with the major-allele reference and the personalized reference at: `https://github.com/alshai/levioSAM/wiki`.

```
bcftools consensus -c <reverse.chain> -f <ref> -o <consensus.fa> <vcf>
awk '{ if ($0 ~ /^chain/) {print} else {print $1, $3, $2} }' <reverse.chain> > <chain>
```

S2 Supplementary Tables

Table S1: Alignment accuracy comparison (Bowtie2). 10M paired-end reads were simulated from the first haplotype of the chr21 genome from NA12878 using mason simulator. Reads were aligned to chr21 of GRCh38, major-allele reference (*Major*) and the same NA12878 genome where reads were simulated from (*Personalized*). Default Bowtie2 parameters were used. The variant-aware reference genomes – *Major* and *Personalized* – used genotypes from the 1000 Genomes Project, including both SNVs and indels. Alignments against the variant-aware reference genomes were processed by levioSAM.

Method	Number of unaligned reads	Fraction of correct reads	Fraction of correct alignments
GRCh38	2183	95.53%	95.54%
Major	975	95.55%	95.55%
Personalized	30	95.56%	95.56%

Table S2: Alignment accuracy comparison (bwa-mem). 10M paired-end reads were simulated from the first haplotype of the chr21 genome from NA12878 using mason simulator. Reads were aligned to chr21 of GRCh38, major-allele reference (*Major*) and the same NA12878 genome where reads were simulated from (*Personalized*). Default bwa-mem parameters were used. The variant-aware reference genomes – *Major* and *Personalized* – used genotypes from the 1000 Genomes Project, inclduing both SNVs and indels. Alignments against the variant-aware reference genomes were processed by levioSAM.

Method	Number of unaligned reads	Fraction of correct reads	Fraction of correct alignments
GRCh38	0	95.54%	95.54%
Major	0	95.55%	95.55%
Personalized	0	95.57%	95.57%

S3 Supplementary Figures

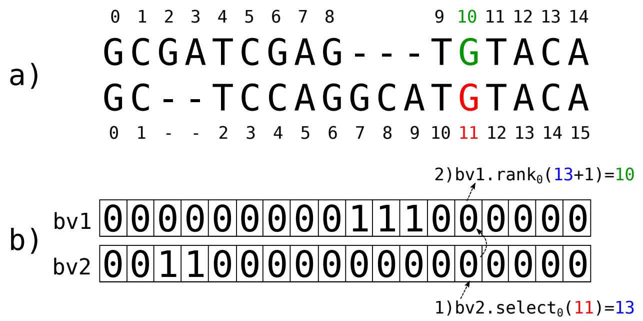

Figure S1: a) Example of a pairwise alignment between two sequences, with offsets labeled. The red position in the bottom sequence corresponds to the green position in the top sequence. b) visual representation of how levioSAM lifts over the red position in the bottom sequence to the top sequence. The pairwise alignment is represented by two bitvectors with `1`s corresponding to gaps. (1) A $\texttt{select}_0$ query is performed on the red position w.r.t `bv2`. (2) This value is used as input to a $\texttt{rank}_0$ query w.r.t `bv1` to obtain the corresponding position on the top sequence.

References

[1] 1000 Genomes Project Consortium et al. A global reference for human genetic variation. *Nature*, 526(7571):68, 2015.

[2] James K Bonfield, John Marshall, Petr Danecek, Heng Li, Valeriu Ohan, Andrew Whitwham, Thomas Keane, and Robert M Davies. Htslib: C library for reading/writing high-throughput sequencing data. *GigaScience*, 10(2):giab007, 2021.

[3] Nae-Chyun Chen, Brad Solomon, Taher Mun, Sheila Iyer, and Ben Langmead. Reference flow: reducing reference bias using multiple population genomes. *Genome biology*, 22(1):1–17, 2021.

[4] Pauline A Fujita, Brooke Rhead, Ann S Zweig, Angie S Hinrichs, Donna Karolchik, Melissa S Cline, Mary Goldman, Galt P Barber, Hiram Clawson, Antonio Coelho, et al. The ucsc genome browser database: update 2011. *Nucleic acids research*, 39(suppl_1):D876–D882, 2010.

[5] Simon Gog, Timo Beller, Alistair Moffat, and Matthias Petri. From theory to practice: Plug and play with succinct data structures. In *13th International Symposium on Experimental Algorithms, (SEA 2014)*, pp. 326–337, 2014.

[6] Manuel Holtgrewe. Mason: a read simulator for second generation sequencing data. *Technical Reports of Institut für Mathematik und Informatik, Freie Universität Berlin*, TR-B-10-06, 2010.

[7] Benjamin Kaminow, Sara Ballouz, Jesse Gillis, and Alexander Dobin. Virtue as the mean: Pan-human consensus genome significantly improves the accuracy of rna-seq analyses. *bioRxiv*, 2020.

[8] Jeremie S Kim, Can Firtina, Meryem Banu Cavlak, Damla Senol Cali, Nastaran Hajinazar, Mohammed Alser, Can Alkan, and Onur Mutlu. Airlift: A fast and comprehensive technique for remapping alignments between reference genomes. *bioRxiv*, 2021.

[9] Ben Langmead and Steven L Salzberg. Fast gapped-read alignment with bowtie 2. *Nature methods*, 9(4):357, 2012.

[10] Heng Li. Aligning sequence reads, clone sequences and assembly contigs with bwa-mem. *arXiv preprint arXiv:1303.3997*, 2013.

[11] Heng Li, Bob Handsaker, Alec Wysoker, Tim Fennell, Jue Ruan, Nils Homer, Gabor Marth, Goncalo Abecasis, and Richard Durbin. The sequence alignment/map format and samtools. *Bioinformatics*, 25(16):2078–2079, 2009.

[12] Jacob Pritt, Nae-Chyun Chen, and Ben Langmead. Forge: prioritizing variants for graph genomes. *Genome biology*, 19(1):220, 2018.

[13] Shuai Yuan and Zhaohui Qin. Read-mapping using personalized diploid reference genome for rna sequencing data reduced bias for detecting allele-specific expression. In *2012 IEEE International Conference on Bioinformatics and Biomedicine Workshops*, pp. 718–724. IEEE, 2012.

[14] Hao Zhao, Zhifu Sun, Jing Wang, Haojie Huang, Jean-Pierre Kocher, and Liguo Wang. Crossmap: a versatile tool for coordinate conversion between genome assemblies. *Bioinformatics*, 30(7):1006–1007, 2014.
